# Palliative radiotherapy for gastric cancer bleeding: a multi-institutional retrospective study

**DOI:** 10.1186/s12904-022-00943-2

**Published:** 2022-04-12

**Authors:** Kazuya Takeda, Toru Sakayauchi, Masaki Kubozono, Yu Katagiri, Rei Umezawa, Takaya Yamamoto, Yojiro Ishikawa, Noriyoshi Takahashi, Yu Suzuki, Keita Kishida, Keiichi Jingu

**Affiliations:** 1 Department of Radiation Oncology, Tohoku University Graduate School of Medicine, 1-1 Seiryo-machi, Aoba-ku, Sendai, Miyagi Japan; 2 Department of Radiation Oncology, Osaki City Hospital, 3-8-1 Furukawa Honami, Osaki, Miyagi Japan; 3 Department of Radiation Oncology, Miyagi Prefecture Cancer Center, 47-1 Shiote Nodayama, Medeshima, Natori, Miyagi Japan; 4 Department of Radiation Oncology, Ishinomaki Red Cross Hospital, 71 Nishimichishita, Hebita, Ishinomaki, Miyagi Japan

**Keywords:** Gastric cancer, Tumor bleeding, Hemostasis, Palliative radiotherapy, Neutrophil-to-lymphocyte ratio (NLR)

## Abstract

**Background:**

Palliative radiotherapy for gastric cancer bleeding has been reported to be a safe and effective treatment, but predictive factors for achievement of hemostasis and overall survival have not been established.

**Methods:**

In this retrospective study, 120 courses of palliative radiotherapy for gastric cancer bleeding in 117 patients in 4 institutes in Japan were reviewed with approval of the ethical committee in each institute. The rate of achieving hemostasis was evaluated by 50% or more reduction of red blood cell transfusion before and after the start of radiotherapy, elevation of blood hemoglobin concentration in a period of 4 weeks from the start of radiotherapy or improvement of subjective or objective clinical symptoms in a period of 4 weeks from the start of radiotherapy. Predictive factors for overall survival and achieving hemostasis were investigated with the Cox hazards model.

**Results:**

The median overall survival period was 3.7 months. Multivariate analysis showed that absence of metastatic disease, higher biological effective dose, higher serum albumin level, lower blood urea nitrogen level and lower neutrophil-to-lymphocyte ratio (NLR) were associated with longer overall survival. Elevation of hemoglobin concentration in a period of 4 weeks from the start of radiotherapy (mean concentration: 8.2 g/dL vs. 8.9 g/dL, *p* = 0.006) and decrease in the amount of red cell transfusion from a 4-week period before to a 4-week period after the start of radiotherapy (mean amount: 716 mL vs. 230 mL, *p* < 0.0001) were observed. The overall rate of achievement of hemostasis was 59.6%. In multivariate analysis, higher biological effective dose was associated with achievement of hemostasis. Grade 2 or higher acute adverse effects related to radiotherapy were observed in 17.5% of cases in 120 treatment courses. Six cases (5.0%) had grade 3 or 4 adverse effects including gastric penetration in 1 patient and anorexia requiring total parental nutrition in 3 patients. No grade 5 adverse effects were observed.

**Conclusions:**

Palliative radiotherapy for gastric cancer bleeding seems to be an effective and safe treatment strategy. Higher treatment dose was associated with longer overall survival and a hemostatic effect. Some hematological parameters may predict overall survival, and they would be helpful for deciding the treatment strategy.

## Background

Gastric cancer is the fifth most common malignancy and the second-most common cause of cancer death worldwide. The incidences of gastric cancer are particularly high in East Asian and South American countries. In Japan, 126,009 people were diagnosed with gastric cancer and 44,192 people died from gastric cancer in 2018 [1].

Radiotherapy for gastric cancer is used in preoperative, postoperative, and palliative situations. Palliative radiotherapy for tumor bleeding has been reported to be a safe and effective treatment [2]. Some studies showed that a higher treatment dose is associated with a better clinical response [3], whereas another study showed that a short course and low dose of radiotherapy were adequate for palliative treatment of bleeding [4]. There has been no randomized controlled trial to determine the optimal treatment dose. In clinical practice, treatment dose is often decided based on the estimated patient’s prognosis with consideration of knowledge about pain control radiotherapy for bone metastases where a smaller dose is known to be related to a higher rate of symptom recurrence. However, there is no established method for predicting the prognosis of patients receiving palliative radiotherapy for gastric bleeding.

In this study, we collected data from multiple centers and investigated the effectiveness of radiotherapy in patients with locally advanced gastric cancer and the prognostic factors in such patients.

## Methods

### Study design

This study was a multi-institutional retrospective study.

### Patients

In this study, the medical records of 117 patients who underwent palliative radiotherapy for bleeding from a gastric neoplasm from September 2014 to March 2021 in 4 institutes in Japan were reviewed. In principle, anticoagulants were stopped before start of radiation therapy.

### Radiotherapy

All patients underwent 3-dimensional conformal radiotherapy (3D-CRT) using computed tomography (CT)-based planning. Measures for respiratory motion using four-dimensional CT were used if possible with consideration of institutional equipment and status of the patient. The definitions of treatment volumes were as follows. Clinical target volume (CTV) was defined as gross tumor volume (GTV) plus a margin of 0 to 1 cm, but setting the whole stomach as CTV was also allowed. Internal target volume (ITV) was defined as CTV plus a margin of 0 to 2 cm with consideration of tumor respiratory motion and available countermeasures. Planning target volume (PTV) was defined as ITV plus a margin of 1 cm. Modification of target volumes with consideration of the status of the patient was allowed. Treatment was delivered with 2 to 4 beams of 6 to 10MV X-rays generated in a linear accelerator. Multiple-field irradiation was used if it was necessary to reduce the dose to the left kidney and bowel. Intensity-modulated radiotherapy (IMRT) was not used in any of the patients. Thirty Gy in 10 fractions delivered over a period of 2 weeks was considered as standard treatment, but physicians decided the treatment dose for each patient with consideration of the patients’ condition, tumor pathology, and treatment history.

### Follow-up and evaluation

Hospital patient records were retrospectively reviewed. Data were collected from patient records up to September 30, 2021. To determine the effectiveness of treatment for tumor bleeding, the amount of red blood cell transfusion, blood hemoglobin concentration, and changes in symptoms were assessed. In Japan, 1 unit of red blood cell transfusion is 140 mL, which is prepared with 200 mL of donated blood. Hemostasis induced by radiotherapy was defined as follows: (1) 50% or greater reduction in the amount of red blood cell transfusion in a period of 4 weeks before and a period of 4 weeks after the start of radiotherapy if the patient received transfusion before the start of radiotherapy, (2) elevation in blood hemoglobin concentration of 1 g/dl or more at 4 weeks after the start of radiotherapy if the patient did not receive red blood cell transfusion in a period of 4 weeks before and after the start of radiotherapy, and (3) any improvement in objective clinical symptoms (e.g., disappearance of melena) and subjective clinical findings (e.g., confirmation of hemostasis by endoscopy). Acute adverse effects associated with radiotherapy in a period of 4 weeks after the end of radiotherapy were recorded according to Common Terminology Criteria for Adverse Events (CTCAE) version 5.0.

### Statistical analysis

All statistical analyses were conducted using JMP Pro version 16.0 (SAS Institute Inc.). Predictive factors for hemostasis were investigated using logistic regression. Overall survival was estimated by the Kaplan-Mayer method, and the Cox hazards model was used for univariate and multivariate analyses. Student’s t-test or Dunnett’s test was used for evaluation of differences in mean values in two groups or multiple groups. A *p* value less than 0.05 was considered to be statistically significant for each analysis.

## Results

A total of 117 patients were enrolled in this study. Three patients underwent two courses of radiotherapy, and 120 courses of treatment were therefore reviewed. Among the 3 patients who underwent a re-irradiation, median interval of two courses of radiotherapy was 141 days and median total BED_10_ was 67 Gy. Patients’ characteristics are shown in Table 1.

**Table 1:** Characteristics of the patients

sex	female	28 (23.3%)
	male	92 (76.7%)
age	[years]	71.0 (43.9—95.8)*
histology	primary gastric adenocarcinoma	106 (88.3%)
	residual stomach cancer	4 (3.3%)
	primary gastric carcinoma, NOS	4 (3.3%)
	esophageal cancer	3 (2.5%)
	metastatic carcinoma	3 (2.5%)
metastatic disease at the time of radiotherapy	yes	91 (75.8%)
	no	28 (23.3%)
	unknown	1 (0.8%)
history of any other treatments	yes	76 (63.3%)
	no	43 (35.8%)
	unknown	1 (0.8%)
serum hemoglobin concentration	[g/dL]	8.2 (4.5—11.8)*
transfusion in last 4 weeks	[mL]	560 (0—3080)*
dose of radiotherapy in BED_10_	[Gy]	39.0 (7.8—60.0)*
concurrent chemotherapy	yes	14 (11.7%)
	no	106 (88.3%)

Values with an asterisk (*) are shown as median values with ranges, *NOS* not otherwise specified, *BED10*: biological effective dose calculated with α/β = 10 Gy

**Fig. 1:**
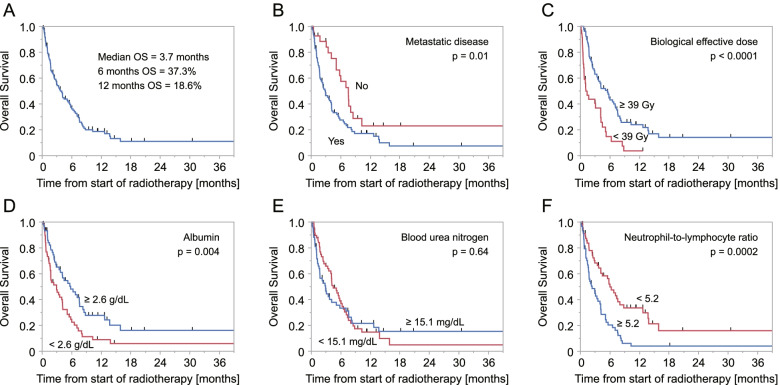
Survival curves plotted by the Kaplan-Meyer method for (**a**) all patients and according to (**b**) existence of metastatic disease, **c** biological effective dose, **d** median value of serum albumin, **e** median value of blood urea nitrogen and *f* median value of neutrophil-to-lymphocyte ratio. Each *p* value was calculated by the log-rank test

Thirty-one patients (26.5%) were alive at the time of data cut-off. The median survival period of the patients was 3.7 months. Survival rates at 6 months and 12 months were 37.3% and 18.6%, respectively (Fig. 1A). We conducted further analyses for prognostic factors for helping the decision of treatment intensity. Table 2 shows the results of univariate and multivariate analyses to determine predictive factors of overall survival. Parameters with *p* values < 0.05 were included into multivariate analysis, in which lymphocyte count and calcium-albumin ratio were excluded as possible confounding factors. In multivariate analysis, absence of metastatic disease, higher biological effective dose, higher serum albumin level, lower blood urea nitrogen level and lower neutrophil-to-lymphocyte ratio (NLR) were associated with longer overall survival. Figure 1B – E show Kaplan-Meyer survival curves according to median values of parameters that were significant in multivariate analysis. As an exploratory analysis, we constructed a formula to predict patients’ survival based on regression coefficients of the pre-treatment parameters.

**Table 2:** Results of univariate and multivariate analysis for predicting overall survival

	univariate analysis		multivariate analysis	
	HR (95% CI)	*p* value	HR (95% CI)	*p* value
Sex (female / male)	1.33 (0.80 – 2.12)	0.25		
age	0.24 (0.20—0.29)	< 0.0001	0.50 (0.14—1.79)	0.28
histology (primary gastric adenocarcinoma / others)	0.76 (0.44 – 1.44)	0.36		
metastatic disease (yes / no)	1.96 (1.17—3.44)	0.01	2.35 (1.27—4.55)	0.01
red blood cell transfusion in last 4 weeks	1.91 (1.63 – 2.24)	< 0.0001	0.74 (0.26—1.89)	0.55
history of any other treatments (yes / no)	1.26 (0.81 – 1.98)	0.31		
biological treatment dose (BED_10_)	0.06 (0.04—0.07)	< 0.0001	0.09 (0.02—0.53)	0.007
concurrent chemotherapy (yes / no)	0.82 (0.40 – 1.51)	0.55		
blood test				
hemoglobin	0.65 (0.52 – 0.81)	0.0001	0.81 (0.21—3.12)	0.76
hematocrit	0.61 (0.16 – 2.18)	0.45		
red blood cell count	0.48 (0.17 – 1.52)	0.21		
white blood cell count	0.60 (0.21 – 1.53)	0.31		
neutrophil count	0.69 (0.23 – 1.83)	0.48		
lymphocyte count	0.39 (0.15—0.92)	0.04	-	-
platelet count	2.45 (1.00—5.47)	0.04	1.79 (0.64—4.67)	0.25
calcium	0.11 (0.01 – 1.17)	0.07		
albumin	0.36 (0.22—0.58)	< 0.0001	0.11 (0.02—0.54)	0.008
blood urea nitrogen	6.42 (1.56—21.87)	0.006	6.51 (1.70—21.78)	0.004
creatinine	0.34 (0.05 – 2.13)	0.27		
total bilirubin	2.68 (0.58 – 7.93)	0.13		
lactate dehydrogenase	6.58 (1.16—23.18)	0.01	3.35 (0.42—15.45)	0.17
C-reactive protein	12.08 (2.32—53.60)	0.002	3.41 (0.47—20.65)	0.20
neutrophil-to-lymphocyte ratio	9.94 (2.84—29.59)	0.0001	6.91 (1.36—29.62)	0.01
platelet-lymphocyte ratio	2.49 (0.67 – 6.79)	0.12		
calcium-albumin ratio	1.84 (1.33—2.57)	0.0003	-	-

*HR* hazard ratio, *CI* confidence interval, *BED10* biological effective dose calculated with α/β = 10 Gy

$$Score=serum albumin level [mg/dL]-0.27 \left(if M1 disease\right)-0.04 * NLR-0.03 * BUN [mg/dL]$$

When patients are divided into 2 groups by the median value of 1.56, median survival periods were 7.1 and1.8 months in higher and lower score group, respectively (*p* < 0.0001 by log-rank test).

**Fig. 2:**
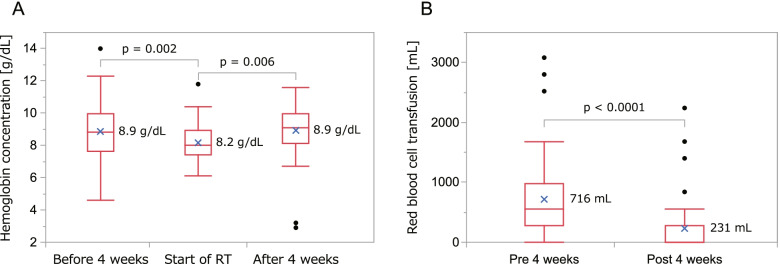
Treatment evaluation. **a** Changes in hemoglobin concentration. **b** Amounts of red blood cell transfusion before and after 4 weeks from start of radiotherapy plotted with box plots. Cross marks show mean values. Dunnett’s test and Students’ t-test were used

**Fig. 3:**
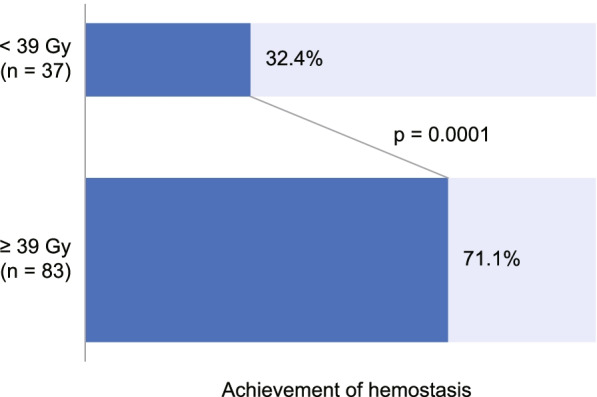
Achievement of hemostasis by treatment dose. Two-sided Fischer’s exact test was used to evaluate the differences in rates of response

In 120 treatment courses, 85 (70.8%) cases could be followed up for 4 weeks after the start of radiotherapy. In those cases, the mean amounts of red blood cell transfusion before and 4 weeks after the start of radiotherapy were 716 mL and 230 mL, respectively (*p* < 0.0001, Fig. 2A). A decrease of more than 50% in the amount of transfusion was achieved in 71% of the 85 cases. The mean hemoglobin concentration at the start of radiotherapy was 8.2 g/dL. Significant decline in the mean hemoglobin concentration was observed in 4 weeks before the start of radiotherapy and significant recovery was observed in 4 weeks after the start of radiotherapy (*p* = 0.002 and 0.006, respectively, Fig. 2B). Subjective and objective improvements of clinical symptoms were accessed in all of the 120 treatment courses, and improvement was achieved in 26 cases (21.7%). Hemostasis was achieved in 59.6% of the 120 cases. Table 3 shows the results of univariate and multivariate analyses to predict achievement of hemostasis. In multivariate analysis, higher biological effective dose was associated with achievement of hemostasis. The rates of hemostasis were 71.1% in patients who received 39 Gy in BED_10_ or a higher dose and 32.4% in patients who received less than 39 Gy (*p* < 0.0001, Fig. 3).

**Table 3:** Results of univariate and multivariate analysis for predicting treatment response

	univariate analysis		multivariate analysis	
	OR (95% CI)	*p* value	OR (95% CI)	*p* value
Sex (female / male)	0.42 (0.18—0.99)	0.05	0.44 (0.17—1.12)	0.08
age	1.44 (0.24—8.64)	0.69		
histology (primary gastric adenocarcinoma / others)	0.78 (0.25—2.50)	0.68		
metastatic disease (yes / no)	0.74 (0.31—1.78)	0.50		
red blood cell transfusion in last 4 weeks	6.17 (0.85—44.81)	0.07		
history of any other treatments (yes / no)	0.91 (0.42—1.94)	0.80		
biological treatment dose (BED_10_)	65.33 (4.38—974.14)	0.002	43.44 (2.48—761.55)	0.01
concurrent chemotherapy (yes / no)	0.91 (0.29—2.81)	0.87		
blood test				
hemoglobin	0.63 (0.08—5.10)	0.66		
hematocrit	0.47 (0.05—4.18)	0.50		
red blood cell count	0.64 (0.08—4.90)	0.67		
white blood cell count	1.28 (0.25—6.61)	0.77		
neutrophil count	1.13 (0.20—6.39)	0.89		
lymphocyte count	1.40 (0.33—5.88)	0.65		
platelet count	0.39 (0.08—1.81)	0.23		
calcium	5.88 (0.13—261.97)	0.36		
albumin	9.57 (1.31—69.82)	0.03	3.68 (0.36—37.19)	0.27
blood urea nitrogen	0.13 (0.01—1.45)	0.10		
creatinine	0.40 (0.03—5.72)	0.50		
total bilirubin	2.65 (0.06—123.70)	0.62		
lactate dehydrogenase	0.05 (0.00—3.61)	0.17		
C-reactive protein	0.04 (0.00—0.81)	0.04	0.16 (0.00—7.72)	0.36
neutrophil-to-lymphocyte ratio	0.42 (0.04—4.95)	0.49		
platelet-lymphocyte ratio	0.19 (0.01—3.68)	0.27		
calcium-albumin ratio	0.38 (0.02—6.02)	0.49		

*OR* odds ratio, *CI* confidence interval, *BED10* biological effective dose calculated with α/β = 10 Gy

Grade 2 or higher acute adverse effects related to radiotherapy occurred in 17.5% of the cases. 6 cases (5.0%) had grade 3 or higher acute adverse effects, including grade 3 anorexia requiring total parenteral nutrition in 5 patients and grade 4 gastric perforation treated with emergency surgery in 1 patient. The patient with grade 4 gastric perforation had a T4 primary region invading the liver and treated with 30 Gy in 10 fractions of radiotherapy. The patient presented a gastric perforation on the next day of completion of the radiotherapy, which resulted in an emergent surgery. No grade 5 adverse effects were observed. Among 3 patients who underwent two courses of gastric irradiation, no grade 2 or higher acute adverse effects were observed.

## Discussion

In this multicenter retrospective study, patients who underwent palliative radiotherapy for gastric neoplasm bleeding were reviewed. Hemostasis was achieved in 59.6% of treatment courses after palliative radiotherapy, and 5.0% of the cases had grade 3 or higher acute adverse effects. One of the largest retrospective studies on palliative radiotherapy for gastric cancer bleeding was carried out by Tey et al. [5]. They analyzed data for 115 advanced symptomatic gastric cancer patients and reported that the response rate to radiotherapy for bleeding was 80.6%, where treatment response was defined as no further blood transfusion or endoscopic treatment in 1 month after treatment. Viani et al. conducted a meta-analysis including 11 studies and reported that the overall response rate for bleeding was 77.7% [3], which is higher than that in the present study. In the present study, some of the patients were transferred to other hospitals immediately after radiotherapy and could not be followed up. This may be associated with the lower rate of hemostasis. In fact, the response rate was 77.8% when we included only 85 patients who could be followed up for more than 4 weeks, and that rate is consistent with the response rates in previous studies. Saito et al. recently published a prospective observational study and evaluated the hemostatic effect in multiple timepoints, where they defined bleeding response as hemoglobin level ≥ 8.0 g/dL, 7 consecutive days without blood transfusion and no salvage therapy for bleeding [6]. They reported that the overall response rate at 4 weeks being 53% and the per-protocol response rate at 4 weeks being 78%, showing the similar tendency to our data [6].

Multivariate analysis in our study showed that higher treatment dose was an independent predictor for achieving a hemostatic effect. For gastric cancer patients, a meta-analysis has shown that 30 Gy or a higher dose in BED_10_ was associated with bleeding response [3]. If the patient is tolerable, higher dose of radiotherapy seems to be beneficial in terms of hemostasis. On the other hand, the efficacy of a low dose and a short course of radiotherapy has been established by randomized control trials in patients with inoperable non-small cell lung cancer [7, 8] and patients with bladder carcinoma [9]. One study showed that a single course or multiple courses of radiotherapy at a dose of 6 Gy in 3 fractions was safe and effective for bleeding gastric carcinoma [4]. Considering these reports, it seems to be reasonable to consider a short course of radiotherapy for some patients with limited prognosis. In the present study, we provided a formula to predict patients’ prognosis. Although this formula needs to be evaluated in another cohort, it might help physicians choose treatment dose and fractionation.

In survival analysis, Treatment BED_10_ was a significant predictor for patients’ survival. However, treatment dose and fractionation are often decided based on physicians’ estimation (i.e. patients with better performance status is tended to receive higher dose of radiotherapy) and this selection bias should be noted. We also revealed some predictive factors besides lower BED_10_ for shorter overall survival including presence of metastatic disease, lower serum albumin concentration, higher blood urea nitrogen level and higher neutrophil-to-lymphocyte ratio. Recently, some hematological parameters have been shown to be related to prognosis in cancer patients treated with radiotherapy [10]. In the present study, among 4 patient parameters (metastatic disease, serum albumin, blood urea nitrogen and NLR), NLR showed the most significant difference in overall survival between two groups. High NLR is regarded to represent systemic inflammatory status and relatively weakened lymphocyte-mediated immune response to tumor. A relationship between high NLR and poor prognosis has recently been shown in various types of cancer [11]. NLR has been reported to be prognostic factor in gastric cancer patients treated with chemotherapy, chemoradiotherapy and surgery [12, 13], but there has been no report for patients treated with palliative radiotherapy. The results of the present study suggest that NLR predicts prognosis and might be helpful for choosing the treatment dose. Serum albumin level was another predictor for patients’ survival in our analysis. The relationship between lower serum albumin level and poor prognosis has been reported in various types of cancer including gastric cancer [14]. Lower serum albumin level is thought to reflect both malnutrition and systemic inflammatory status. This study also revealed that higher BUN level was related to shorter overall survival. BUN is not a common prognostic factor in cancer patients, but it is reported that increased BUN is related to high mortality rate in patient with gastrointestinal bleeding in emergency department [15]. High BUN level may indicate an advanced primary tumor through increased nitrogen intake caused by gastrointestinal bleeding.

In the present study, grade 3 or higher acute adverse effects occurred in 6 patients (5.0%). Five of those patients had grade 3 anorexia and 1 patient had grade 4 gastric perforation. Considering the site of disease, perforation seems to be an inevitable adverse effect to some extent. Overall, the rate of acute adverse effects seemed to be acceptable.

We recognize some limitations in our study. First, there were data defects because of the study being a retrospective multicenter study. We could not acquire follow-up data for some patients who were transferred to other hospitals soon after completion of radiotherapy. Secondly, the treatment dose depended on the physicians’ choice, and that bias makes it difficult to evaluate the relationship between treatment dose and hemostatic effect. Thirdly, most of the patients were treated with a dose of 30 Gy in 10 fractions (64.2%) or a dose of 20 Gy in 5 fractions (19.2%), and it was therefore difficult to examine the efficacy of single-dose radiotherapy or high-dose radiotherapy over 39 Gy in BED_10_.

## Conclusions

In conclusion, palliative radiotherapy for gastric cancer bleeding seems to be an effective and relatively safe treatment strategy. A dose of 30 Gy in 10 fractions or a higher dose might be associated with a better hemostatic effect.

## Data Availability

The datasets used and/or analyzed during the current study are available from the corresponding author on reasonable request.

## Abbreviations

3D-CRT: 3-Dimensional conformal radiotherapy
CT: Computed tomography
CTV: Clinical target volume
GTV: Gross tumor volume
ITV: Internal target volume
PTV: Planning target volume
IMRT: Intensity-modulated radiotherapy
CTCAE: Common Terminology Criteria for Adverse Events
NOS: Not otherwise specified
BED_10_: Biological effective dose calculated with α/β = 10 Gy
